# Supplementary material for: Cat caregivers’ perceptions, motivations, and behaviours for feeding treats: A cross sectional study
Source: PLoS One. 2024 Jan 10;19(1):e0296011. doi: 10.1371/journal.pone.0296011 (PMC10781132; doi:10.1371/journal.pone.0296011)
Supplement: S1 Appendix — (PDF) [file pone.0296011.s001.pdf]

Pet Treat Survey Questions

**This is a PDF version of a larger Qualtrics online survey designed to collect data on dog and cat caregivers' perceptions and behaviours surrounding treat feeding. Question items that specifically pertained to cat caregivers' perceptions, motivations and decisions about feeding treats are included here.

I identify my gender as:

- Man
- Non-binary
- Woman
- Prefer to self-describe, please specify
  ____

What is your age group?

- 18-25
- 26-35
- 36-45
- 46-59
- 60+
- Prefer not to answer

What is your Country of residence?

▼ Afghanistan ... Zimbabwe

What is the highest degree or level of education you have completed?

- Some high school
- High school
- Apprenticeship training and trades
- College
- Bachelor's degree
- Master's degree
- Professional Degree
- Ph.D. or higher
- Prefer not to answer

Would you consider yourself to have a career related to companion animal care? (e.g. veterinarian, trainer, breeder)

- Yes (please specify) ____
- No
- Prefer not to answer

How many other people live in your household?

- None, just myself
- 1
- 2-4
- More than 4
- Prefer not to answer

How many other people in your household contribute to your pet's care on a regular basis?

- None, just myself
- 1
- 2-4
- More than 4
- Prefer not to answer

In total, how many cats live in your household?

- None
- 1
- 2
- 3+
- Prefer not to answer

In total, how many dogs live in your household?

- None
- 1
- 2
- 3+
- Prefer not to answer

If you are an owner of multiple pets, please answer the remainder of the survey questions with respect to one animal only. If you were to put your pets' names into alphabetical order (from A to Z) pick the pet whose name starts with the letter closest to A.

What species of pet are you answering questions about?

- Cat
- Dog

What is the sex of your pet?

- Male intact
- Male neutered
- Female intact
- Female spayed
- Other, please specify ____

What is the age of your pet?

- <1 year
- 1-3 years
- 4-6 years
- 7+ years

Which diagram best illustrates the body condition of your pet?

[Note: Images removed due to copyright restrictions]

- Image:BCS 3
- Image:BCS 1
- Image:BCS 5
- Image:BCS 4
- Image:BCS 2
- Unsure

Does your pet have any existing health concerns?

- Yes (please specify) ____
- No
- Unsure

Select the statement that best describes the relationship you have with your pet

- Like a child
- Part of my family
- Teammate/partner (i.e. working relationship)
- Companion
- Acquaintance
- Unsure

Please rate the following

	Not attached	Extremely attached
	0 1 2 3 4 5 6 7 8 9 10	
How attached are you to your pet? 0 being not attached, 10 being extremely attached		

What is your pet's diet? Select all that apply

- Dry food/kibble
- Canned/wet food
- Commercial raw diet
- Homemade raw diet
- Homemade cooked diet
- Therapeutic/prescription diet
- Other (please specify) ____

How frequently do you feed your pet's meals?

- Specific amount once daily
- Specific amount twice daily
- Specific amount three times daily
- Always food available (free feeding)
- Other, please specify ____

How do you measure your pet's meals?

- Eyeball estimate
- Measuring cup/scoop
- Random cup/container
- Gram scale
- Servings are pre-portioned when purchased
- I do not measure my pet's meals
- Other, please specify ____

How do you decide on the amount of food to feed your pet? Select all that apply

- I follow the recommended daily feeding amount on the package label
- Veterinary advice
- My own research
- I regularly monitor my pet's weight/body condition
- Based on my pet's recent activity level
- Until my pet seems satisfied
- I have no specific strategy on how much I feed
- Other, please specify ____

How often do you feed your pets' meals using the following modes of delivery?

	Always	Very often	Sometimes	Rarely	Never
Traditional food bowl	○	○	○	○	○
Interactive puzzle feeder/slow feeder/food dispense ball	○	○	○	○	○
Electronic/smart feeder	○	○	○	○	○
Hand feeding	○	○	○	○	○
Stuffed toys (e.g. Kong, West Paw Toppl)	○	○	○	○	○
Other, please specify	○	○	○	○	○

Select the statement that best defines what the term 'treat' means to you

- Anything I give or do with my pet that they enjoy (e.g. new toy, food they enjoy, going for a special walk)
- Any type of food I give my pet that they enjoy (e.g. table scraps, cheese, homemade biscuits, training treats)
- Products sold exclusively as treats for pets (i.e. commercial pet treats only)
- Other, please specify ____

Note: from this point forward, the term 'treat' will only refer to food

Which of the following do you consider to be (food) treats for pets? Select all that apply

- Pet food given outside of regular mealtimes (e.g. kibble used as a reward during training)
- Commercial pet treats (e.g. soft training treats, biscuits)
- Commercial dental treats (e.g. Pedigree Dentastix, Greenies)
- Natural chews (e.g. raw hide, bully sticks)
- Bones (raw or cooked)
- Human food prepared specifically for pet (e.g. fruit/veggies, cheese, dehydrated food items)
- Table scraps
- Fast food
- Food used to disguise medication (e.g. pill pockets, cheese)
- Other, please specify ____

How frequently do you feed your pet treats for the following reasons?

	Always	Very often	Sometimes	Rarely	Never
To reward good behaviour	◯	◯	◯	◯	◯
To reward my pet during training/sports activities	◯	◯	◯	◯	◯
Because my pet enjoys them	◯	◯	◯	◯	◯
To keep my pet busy	◯	◯	◯	◯	◯
As a source of enrichment for my pet	◯	◯	◯	◯	◯
Because they contain certain ingredients that are beneficial to my pet's health	◯	◯	◯	◯	◯
To clean my pet's teeth	◯	◯	◯	◯	◯
To disguise my pet's medication	◯	◯	◯	◯	◯
Feelings of guilt (e.g. pet was home alone all day)	◯	◯	◯	◯	◯
To make up for a 'bad' day/event (e.g. vet visit, groom, etc.)	◯	◯	◯	◯	◯
To show my pet that I love them!	◯	◯	◯	◯	◯
Part of a routine	◯	◯	◯	◯	◯
Because I feel like it	◯	◯	◯	◯	◯

How does feeding a treat to your pet make you feel?

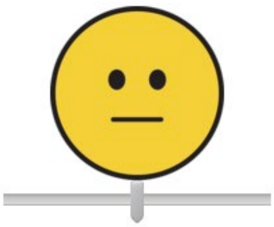

How does your pet react when you feed them treats?

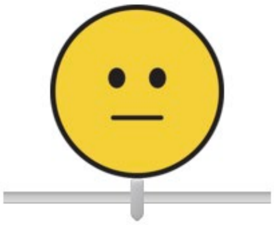

What motivates you to feed your pet treats? Select all that apply

- My pet anticipates treats as part of their routine
- When it appears that my pet asks for treats
- Reinforcing desired behaviour from my pet
- It makes me happy
- It makes my pet happy
- It strengthens the bond with my pet
- Nutritional/health benefits for my pet
- To enrich my pet's day
- To keep my pet busy/occupied
- Unsure
- Other, please specify ____

When thinking about different *types* of treats (e.g. training treats vs. dental treats vs. chews, etc.), how many types of treats do you feed your pet?

- 1 only
- 2
- 3-5
- 6-10
- Too many to count!
- Unsure

What motivates you to feed different *types* of treats? Select all that apply

- I only feed one type of treat to my pet
- To introduce variety to my pet
- To incorporate different ingredients into my pet's diet
- Each treat serves a different purpose (i.e., one is for training, another is to clean teeth, etc.)
- What I purchase or feed depends on what is on sale or recommended to me at the time
- To enrich my pet's life
- No particular reason
- Other, please specify ____

How many different *brands* of the same type of treat (e.g. biscuits, training treats) do you feed your pet?

- 1 only
- 2
- 3-5
- 6-10
- Too many to count!
- Unsure

What motivates you to feed different *brands* of the same type of treats to your pet? Select all that apply

- I only feed one brand of treat to my pet
- To introduce variety to my pet
- To incorporate different ingredients into my pet's diet
- To offer treats of different value to my pet (i.e., higher levels of reward)
- What I purchase or feed depends on what is on sale or recommended to me at the time
- To enrich my pet's life
- No particular reason
- Other, please specify ____

How many different *flavour varieties* of the same type of treat (e.g. biscuits, training treats) do you feed your pet?

- 1 only
- 2
- 3-5
- 6-10
- Too many to count!
- Unsure

What motivates you to feed different *flavour varieties* of the same type of treats? Select all that apply

- I only feed one flavour variety of treat to my pet
- To introduce variety to my pet
- To incorporate different ingredients into my pet's diet
- To offer treats of different value to my pet (i.e., higher levels of reward)
- What I purchase or feed depends on what is on sale or recommended to me at the time
- To enrich my pet's life
- No particular reason
- Other, please specify ____

What factors are likely to influence what treats you feed to your pet?

	Very likely	Likely	Neither likely/unlikely	Unlikely	Very unlikely
Main ingredient (e.g. chicken, beef, vegetable)	○	○	○	○	○
Complete ingredient composition	○	○	○	○	○
Shape (e.g. cubed, braided, hearts)	○	○	○	○	○
Size of treat	○	○	○	○	○
Health claims (e.g. freshens breath, presence of omega 3)	○	○	○	○	○
Moisture content/texture of treat	○	○	○	○	○
Price	○	○	○	○	○
Brand	○	○	○	○	○
Origin (e.g. made in Canada)	○	○	○	○	○
Taste (I know my pet likes it)	○	○	○	○	○
Veterinary recommendation	○	○	○	○	○
Pet store sales associate recommendation	○	○	○	○	○
Online recommendation	○	○	○	○	○
Friend/Family/Coworker recommendation	○	○	○	○	○

Where do you source the treats that you feed your pet? Select all that apply

- Food that we already have around the house (e.g., cheese, fruit/veggies)
- Tables scraps/leftovers
- Fast food establishments
- I make my own pet treats at home (e.g. bake my own pet biscuits, dehydrate fruit/vegetables/meat)
- I use my pet's food as treats (e.g. their kibble)
- I buy commercial pet treats from the grocery store
- I buy commercial pet treats from a pet store/online pet retailer
- I buy treats from my veterinary clinic
- I source ingredients directly from a butcher/farmer's market
- Other, please specify ____

How often do you feed your pet the following:

	Daily	A few times a week	Weekly	Monthly	Never
Cookies/biscuits	○	○	○	○	○
Soft and chewy/training treats	○	○	○	○	○
Jerky	○	○	○	○	○
Chews (e.g. bully sticks, raw hide)	○	○	○	○	○
Bones	○	○	○	○	○
Dental treats	○	○	○	○	○
Human food (prepared specifically for pet)	○	○	○	○	○
Table scraps	○	○	○	○	○

Do you monitor your pet's treat intake?

- Yes
- No
- Sometimes

How do you measure your pet's treats? Select all that apply

- Eyeball estimate
- Measuring cup/scoop
- Random cup/container
- Gram scale
- Servings are pre-portioned when purchased (e.g. sample bag of treats, bully stick, etc.)
- I do not measure my pet's treats
- Other, please specify ____

How do you decide on the amount of treats to feed your pet? Select all that apply

- I follow the recommended daily feeding amount on the package label
- Veterinary advice
- My own research
- I regularly monitor my pet's weight/body condition
- Based on my pet's recent activity level
- Until my pet seems satisfied
- No specific reason
- Other, please specify ____

How would you consider your pet's treats in relation to their normal diet?

- Treats are part of my pet's normal diet
- Some treats are part of my pet's normal diet, while others are an additional extra
- Treats are an additional extra to my pet's normal diet
- Unsure

Approximately what percent (0-100%) of your pet's diet derives from treats, based on estimated quantity?

	0	25	50	75	100
%					

How often do you feed your pet treats using the following modes of delivery?

	Always	Very often	Sometimes	Rarely	Never
Traditional food bowl	○	○	○	○	○
Interactive puzzle feeder/slow feeder/food dispense ball	○	○	○	○	○
Electronic/smart feeder (e.g., Furbo)	○	○	○	○	○
Hand feeding	○	○	○	○	○
Stuffed toys (e.g. Kong, West Paw Toppl)	○	○	○	○	○
Other, please specify	○	○	○	○	○
