# Supplementary material for: Cat caregivers’ perceptions, motivations, and behaviours for feeding treats: A cross sectional study
Source: PLoS One. 2024 Jan 10;19(1):e0296011. doi: 10.1371/journal.pone.0296011 (PMC10781132; doi:10.1371/journal.pone.0296011)
Supplement: Attachment — Submitted filename: Response to Reviewers.docx [file pone.0296011.s002.docx]

Response to Reviewer comments:

PONE-D-23-20840

Cat caregivers’ perceptions, motivations, and behaviours for feeding treats: a cross sectional study

PLOS ONE

Dear Dr. Nielson,

Thank you for submitting your manuscript to PLOS ONE. After careful consideration, we feel that it has merit but does not fully meet PLOS ONE’s publication criteria as it currently stands. Therefore, we invite you to submit a revised version of the manuscript that addresses the points raised during the review process.

Please submit your revised manuscript by Dec 07 2023 11:59PM. If you will need more time than this to complete your revisions, please reply to this message or contact the journal office at [plosone@plos.org](mailto:plosone@plos.org). When you're ready to submit your revision, log on to <https://www.editorialmanager.com/pone/> and select the 'Submissions Needing Revision' folder to locate your manuscript file.

Please include the following items when submitting your revised manuscript:

- A rebuttal letter that responds to each point raised by the academic editor and reviewer(s). You should upload this letter as a separate file labeled 'Response to Reviewers'.
- A marked-up copy of your manuscript that highlights changes made to the original version. You should upload this as a separate file labeled 'Revised Manuscript with Track Changes'.
- An unmarked version of your revised paper without tracked changes. You should upload this as a separate file labeled 'Manuscript'.

If you would like to make changes to your financial disclosure, please include your updated statement in your cover letter. Guidelines for resubmitting your figure files are available below the reviewer comments at the end of this letter.

If applicable, we recommend that you deposit your laboratory protocols in protocols.io to enhance the reproducibility of your results. Protocols.io assigns your protocol its own identifier (DOI) so that it can be cited independently in the future. For instructions see: <https://journals.plos.org/plosone/s/submission-guidelines#loc-laboratory-protocols>. Additionally, PLOS ONE offers an option for publishing peer-reviewed Lab Protocol articles, which describe protocols hosted on protocols.io. Read more information on sharing protocols at <https://plos.org/protocols?utm_medium=editorial-email&utm_source=authorletters&utm_campaign=protocols>.

We look forward to receiving your revised manuscript.

Kind regards,

Harvie P. Portugaliza, D.V.M., Ph.D.
Academic Editor
PLOS ONE

Journal Requirements:

When submitting your revision, we need you to address these additional requirements.

1. Please ensure that your manuscript meets PLOS ONE's style requirements, including those for file naming. The PLOS ONE style templates can be found at 
<https://journals.plos.org/plosone/s/file?id=wjVg/PLOSOne_formatting_sample_main_body.pdf> and 
<https://journals.plos.org/plosone/s/file?id=ba62/PLOSOne_formatting_sample_title_authors_affiliations.pdf>

*Response: Thank you, we have carefully reviewed these templates to ensure the manuscript meets PLOT ONE’s style requirements.*

2. Thank you for stating the following in the Competing Interests section: 
"A.V. is the Royal Canin Veterinary Diets Endowed Chair in Canine and Feline Clinical Nutrition and declares that they serve on industry advisory boards and received honoraria and research funding from various pet food manufacturers and ingredient suppliers."

Please confirm that this does not alter your adherence to all PLOS ONE policies on sharing data and materials, by including the following statement: ""This does not alter our adherence to  PLOS ONE policies on sharing data and materials.” (as detailed online in our guide for authors <http://journals.plos.org/plosone/s/competing-interests>).  If there are restrictions on sharing of data and/or materials, please state these. Please note that we cannot proceed with consideration of your article until this information has been declared.

Please include your updated Competing Interests statement in your cover letter; we will change the online submission form on your behalf.

*Response: Thank you, we have updated a Competing Interests statement in the cover letter.*

3. We note that you have stated that you will provide repository information for your data at acceptance. Should your manuscript be accepted for publication, we will hold it until you provide the relevant accession numbers or DOIs necessary to access your data. If you wish to make changes to your Data Availability statement, please describe these changes in your cover letter and we will update your Data Availability statement to reflect the information you provide.

*Response: The authors do not declare any changes to the Data Availability Statement at this time. We confirm that repository information for the data will be provided upon acceptance.*

4. We note that Figures S1 Appendix in your submission contain copyrighted images. All PLOS content is published under the Creative Commons Attribution License (CC BY 4.0), which means that the manuscript, images, and Supporting Information files will be freely available online, and any third party is permitted to access, download, copy, distribute, and use these materials in any way, even commercially, with proper attribution. For more information, see our copyright guidelines: <http://journals.plos.org/plosone/s/licenses-and-copyright>.
 
We require you to either (1) present written permission from the copyright holder to publish these figures specifically under the CC BY 4.0 license, or (2) remove the figures from your submission:
 
a. You may seek permission from the original copyright holder of Figurs S1 Appendix to publish the content specifically under the CC BY 4.0 license. 
 
We recommend that you contact the original copyright holder with the Content Permission Form (<http://journals.plos.org/plosone/s/file?id=7c09/content-permission-form.pdf>) and the following text:
“I request permission for the open-access journal PLOS ONE to publish XXX under the Creative Commons Attribution License (CCAL) CC BY 4.0 (<http://creativecommons.org/licenses/by/4.0/>). Please be aware that this license allows unrestricted use and distribution, even commercially, by third parties. Please reply and provide explicit written permission to publish XXX under a CC BY license and complete the attached form.”
 
Please upload the completed Content Permission Form or other proof of granted permissions as an ""Other"" file with your submission. 
 
In the figure caption of the copyrighted figure, please include the following text: “Reprinted from [ref] under a CC BY license, with permission from [name of publisher], original copyright [original copyright year].”
 
b. If you are unable to obtain permission from the original copyright holder to publish these figures under the CC BY 4.0 license or if the copyright holder’s requirements are incompatible with the CC BY 4.0 license, please either i) remove the figure or ii) supply a replacement figure that complies with the CC BY 4.0 license. Please check copyright information on all replacement figures and update the figure caption with source information. If applicable, please specify in the figure caption text when a figure is similar but not identical to the original image and is therefore for illustrative purposes only.

*Response: Thank you for bringing this important information to our attention. We have decided to remove the figure and included a statement to reflect this in the “S1 Appendix” document that presents the questions used for data collection. Instead, we have clarified in the methodology section that the images presented resembled those presented from the 5-point body condition score presented by the American Animal Hospital Association and have referenced this material for readers.*

5. Please include captions for your Supporting Information files at the end of your manuscript, and update any in-text citations to match accordingly. Please see our Supporting Information guidelines for more information: <http://journals.plos.org/plosone/s/supporting-information>.

*Response: a caption for the Supporting Information has been added to the end of the manuscript (lines 815-817).*

6. Please review your reference list to ensure that it is complete and correct. If you have cited papers that have been retracted, please include the rationale for doing so in the manuscript text, or remove these references and replace them with relevant current references. Any changes to the reference list should be mentioned in the rebuttal letter that accompanies your revised manuscript. If you need to cite a retracted article, indicate the article’s retracted status in the References list and also include a citation and full reference for the retraction notice.

*Reviewers' comments: the authors have reviewed the reference list to ensure accuracy and completeness.*

Reviewer's Responses to Questions

**Comments to the Author**

1. Is the manuscript technically sound, and do the data support the conclusions?

The manuscript must describe a technically sound piece of scientific research with data that supports the conclusions. Experiments must have been conducted rigorously, with appropriate controls, replication, and sample sizes. The conclusions must be drawn appropriately based on the data presented.

Reviewer #1: Yes

Reviewer #2: Yes

2. Has the statistical analysis been performed appropriately and rigorously?

Reviewer #1: Yes

Reviewer #2: Yes

3. Have the authors made all data underlying the findings in their manuscript fully available?

The [PLOS Data policy](http://www.plosone.org/static/policies.action#sharing) requires authors to make all data underlying the findings described in their manuscript fully available without restriction, with rare exception (please refer to the Data Availability Statement in the manuscript PDF file). The data should be provided as part of the manuscript or its supporting information, or deposited to a public repository. For example, in addition to summary statistics, the data points behind means, medians and variance measures should be available. If there are restrictions on publicly sharing data—e.g. participant privacy or use of data from a third party—those must be specified.

Reviewer #1: Yes

Reviewer #2: Yes

4. Is the manuscript presented in an intelligible fashion and written in standard English?

PLOS ONE does not copyedit accepted manuscripts, so the language in submitted articles must be clear, correct, and unambiguous. Any typographical or grammatical errors should be corrected at revision, so please note any specific errors here.

Reviewer #1: Yes

Reviewer #2: Yes

5. Review Comments to the Author

Please use the space provided to explain your answers to the questions above. You may also include additional comments for the author, including concerns about dual publication, research ethics, or publication ethics. (Please upload your review as an attachment if it exceeds 20,000 characters)

Reviewer #1: This article is indispensable in addressing the questions on the role of cat owners and caregivers toward feline nutrition. It contributes knowledge for a comprehensive understanding of the factors surrounding feline nutrition, particularly human behavior. The authors gave adequate background information on the prevalence of feline obesity, its comorbidities, and the role of treat feeding among other factors. The researchers attempted to address the question relating to cat caregivers’ behaviors, perceptions, and decisions in providing treats to their cats by conducting a cross-sectional survey and analyzing the data to identify significant factors. The authors found out that caregivers perceive treat as anything that their pet enjoys, including food items (i.e., commercial pet treats), and are considered part of the normal feline diet. Most of the caregivers selected the type of treats primarily based on the taste (knowing their pet likes it). Friends, family, coworkers, and online recommendations; as well as price and brand significantly influence younger caregivers in selecting the type of treats. Decisions on the amount of treats given to their pets were made mainly based on the body condition score (BCS). Middle-aged caregivers rely more on veterinary advice, while caregivers in younger age groups rely on their own research. In general, the article provides valuable insights into the perceptions, motivations, and behaviors of cat caregivers surrounding treat feeding, which is an area worth exploring. I recommend publishing the article.
The main strength of the article is that it addresses the questions on social aspects relating to feline nutrition and to the prevalence of feline obesity by providing clear information on the behaviors, perceptions, and motivations of cat caregivers. Importantly, the article conforms to the STROBE checklist.

*Response: Thank you very much! We appreciate these comments and support of the article.*

Weaknesses of the article are well stated in the discussion section such as potential bias due to the use of convenience sampling and self-selection in the study, and the results may only reflect a subpopulation of caregivers. Nevertheless, the authors stipulated it to give the readers a precaution for interpreting the results. There is also a lack of evidence for internal consistency for Likert-scale questions in the questionnaire used in the study. I suggest further revision of the article as there are minor typographical errors and needed improvements and clarifications.
For the improvement of the article, the authors are encouraged to resolve the following minor issues:

1. In line 33 the authors claim the model is predictive, however, there are no mention of meeting assumption requirements. Hence it is suggested that the authors perform assumption checks (i.e. multicollinearity, VIF, etc.) and mentioned in the data analysis section. The overall model p-value should be reported in the multivariable tables. To further know how well the logistic regression model classifies the outcome, predicted probabilities can be used against the dependent variable to make an ROC curve and the area under the curve can be reported (Homer and Lemeshow, 2000).

*Response: Thank you very much for these suggestions and helpful references. We have reported the overall p-values for each model in Table 5. Furthermore, we expanded on the methodology section to report additional assumption checks using VIF, and area under the ROC curve was used to evaluate how well the logistic regression models classified the outcomes (lines 217-220).*

2. The authors may also mention estimates of the prevalence of obesity in domestic cats in Canada in the second paragraph of the Introduction section.

*Response: The authors were unable to find a recent estimate of the prevalence of overweight and obesity in domestic cats in Canada specifically, so we have included a few recent estimates of overweight and obesity from domestic cats in the U.S. and generalized these findings to North American populations (line 50).*

3. Typo error in line 64 “white”.

*Response: This error has been corrected.*

4. In Line 75-78 the authors described “caregiver”. The authors should clarify if this is different from cat owners especially that in Table 1 other people in household are considered.

*Response: We have clarified that the term “caregiver” is considered synonymous to “cat owner” (lines 80-81). Participants self-identified as a primary caregiver of at least one cat in this study. The authors understand that more than one person in the household may also be considered caregivers. This is what we aimed to capture when asking respondents to report 1) the number of other people living in the household in total, and 2) the number of other people living in the household that contribute to the pet’s care (Table 1). These terms were used instead of family members, to capture a broad range of living situations such as roommates who may live with the primary caregiver, but do not provide direct care for the animal. We included a comment about this in the manuscript (lines 108-111) and hope that this provides some clarification.*

5. The authors should mention the questionnaire assessment and its results in data analysis section, e.g. Cronbach’s alpha test for Likert-scale questions

*Response: we have included a statement about questionnaire assessment and results of Cronbach’s alpha test for internal consistency (lines 99-101; 102-104)*

6. The authors should clarify lines 147-148 where it mentions differences in means when in fact the preceding sentences mention categorical variables.

*Response: we have removed “for differences in means” to resolve this (line 166).*

7. The authors should present the mean or median and SD or IQR for continuous variables in Table 1 in line 201.

*Response: There were no continuous variables presented in Table 1. These variables were explored categorically, as presented. As such, we have not included these data points in Table 1. We have clarified the methodology section to clarify the data points presented in the case of mean or median data (lines 153-154). Since all continuous data were not normally distributed, median and range data were presented.*

8. In Table 1, the authors only considered the “other people” living with the family. I think there is a need to consider for the family members because it’s not only the “other people” that contribute to pet’s care but the members of the family as well. The number of household members should have been relevant. The authors should clarify this.

*Response: Thank you for bringing this point to our attention. As previously stated, the wording surrounding “other members of the household” was meant to inclusively capture potential family members, as well as other individuals who may be considered members of the household. To help clarify this, we have included a statement in the methodology section (lines 108-111).*

9. In line 209, the n cats is only equal to 337 the same as the number of respondents. The authors should clarify if only 1 cat is considered per respondent. According to Table 1, the number of cats per household interviewed differed. For example, for 188 households there is 1 cat so 188 in total, 2 cats in 101 households, and 3 cats in 48 households so the total number of cats in the whole study should be = 534. The authors should give light to this discrepancy.

*Response: In the case that respondents identified as the owner of multiple pets, they were asked to complete the survey with respect to one animal only. This question is shown in ’S1 Appendix’ document (provided as supplementary material) that lists the questions that were used for data analysis, and we have included a statement about this in the methodology section to convey this (lines 107-108). As such there were 337 total respondents, and subsequently data provided in regards to 337 cats.*

10. Typo error in Line 225 and 234, Table 3, instead of Table 4.

*Response: Thank you for catching this error, Table 4 has been retitled to Table 3 and referenced as so. Table 3 has been renamed and referenced as Table 4.*

11. The author should state the abbreviation fully in Line 424 if first mentioned.

*Response: Thank you for bringing this to our attention, we have defined the abbreviation (lines 490-491).*

12. Typo error in Line 488.

*Response: The authors appreciate the opportunity to resolve any errors, though we have carefully reviewed line 488 and are not able to identify a typo. Could you please highlight the typo you are referring to specifically?*

References
Hosmer Jr., D.W and Lemeshow, S. (2000) Applied logistic regression. 2nd Edition, John Wiley & Sons, Inc., New York. <http://dx.doi.org/10.1002/0471722146>

Reviewer #2: Broad comments
A well-written manuscript illustrating valuable research aiming to understand perceptions and motivations surrounding provisioning treats to cats.

*Response: Thank you very much!*

Specific comments

L41: remove ‘including’

*Response: We have removed ‘including’ from this sentence (line 41).*

L64: Perhaps a comment could be made that treat feeding is often perceived as a providing a reward and used during positive reinforcement training. There has been some research on this in dogs/cats, with the notion of improving their behavior and often used by owners with a stronger bond.

*Response: We agree that these aspects would be valuable to include, and have commented on them in the Introduction section (lines 69-72).*

L164: stay consistent with terminology, it flows easier for the reader who may not be familiar with this analysis, if you describe the frequency of feeding treats as (explanatory dichotomous yes/no variables) like you explained the other model’s variables.

*Response: Thank you for this suggestion, we have replaced the description of frequency of feeding treats to reflect this (line 187).*

L173: revise: the ‘following’ categorical variables were included in this analysis due to their suspected role… : caregiver age … ,..

*Response: We have revised this sentence as suggested (lines 195-197).*

L217: how was this measured? Was this created for the survey? Did you use a certain scale? Lexington attachment to pets scales? Was this not used as a predictor to determine if it was related to their provision of treats (with the hypothesis that owners with a stronger bond are more likely to feed treats (regardless of BCS))? This would be interesting to explore as though we know cat obesity is such an immense welfare issue, and its often present within households that report a strong bond. I encourage exploring this relationship between attachment and BCS and their influence on provision of treats.

*Response: While we agree that exploring specific aspects related to relationships and level of attachment would be interesting to explore, the goal of our study was to explore caregivers’ perceptions and behaviours related to feeding treats. Being mindful of the length of the questionnaire for respondents and maintaining the primary focus on aspects related to treats specifically, we opted to explore these aspects with two questions developed specifically for the questionnaire. In relation to attachment, we asked respondents, “how attached are you to your pet? 0 being not attached, 10 being extremely attached”. These questions can be viewed in the ‘S1 Appendix’ document, which has been provided to include as supplementary information. Our results found that 84% of respondents rated their level of attachment as ≤ 8/10, indicating a high level of attachment, and therefore level of attachment data is quite skewed which impacts exploration of this variable with the amount of treats provided and makes it difficult to draw meaningful conclusions. Nevertheless, we have expanded the analyses to further report on significant findings in differences of estimated percent of diet composed of treats by reported level of attachment. These findings are presented in Table 6 (new, line 388-389) and elsewhere in the Results section (lines 380-387). We have also commented on these findings in the discussion section (lines 529-538). Inclusion of LAPs in questionnaires exploring treat feeding would be a good opportunity for future research projects.*

Table 5: were all partial f-tests for all these variables presented here significant? Would be useful to add these pvalues above the individual level pvalues (same row as the variable label).
- Method of measurement for treats – what was the referent?
- Feeding frequencies for treats – what was the referent?

*Response: To the author’s knowledge partial F-tests are limited to linear regression. Though we have provided the p-values from likelihood ratio tests, which can also be used to test the contribution of any subset of parameters in the way that partial F-tests can be used in linear regression [1].The variables “method of measurement for treats” and “feeding frequencies for treats”, and their respective categories were explored as individual variables, since respondents had the opportunity to select more than one response option for these questions. We have clarified this in the footnotes of Table 5 (lines 360-364).*

References:

1.	Dohoo I, Martin W, Stryhn H. Veterinary Epidemiologic Research. 2nd Edition ed. Charlottetown, Prince Edward Island, Canada: VER Inc.; 2014.
